# Carbonic Anhydrase I Is Recognized by an SOD1 Antibody upon Biotinylation of Human Spinal Cord Extracts

**DOI:** 10.3390/ijms11104051

**Published:** 2010-10-20

**Authors:** Jian Liu, Armin Akhavan, Mengde Lu, Arie Gruzman, Vishwanath R. Lingappa, Jiyan An, Robert Bowser

**Affiliations:** 1 Department of Neuroscience, California Pacific Medical Center Research Institute, 475 Brannan Street, San Francisco, CA 94107, USA; E-Mail: lumz@cpmcri.org (M.L.); 2 Department of Cancer Research, California Pacific Medical Center Research Institute, 475 Brannan Street, San Francisco, CA 94107, USA; E-Mail: akhavaA@cpmcri.org (A.A.); 3 Department of Bioconformatics, California Pacific Medical Center Research Institute, 475 Brannan Street, San Francisco, CA 94107, USA; E-Mails: lev.gruzman@mail.huji.ac.il (A.G.); vlingappa@prosetta.com (V.R.L.); 4 Department of Pathology, University of Pittsburgh School of Medicine, Pittsburgh, PA 15261, USA; E-Mail: jia4+@pitt.edu (J.A.)

**Keywords:** mass spectrometry, proteomics, biotinylation, SOD1, ALS, carbonic anhydrase I

## Abstract

We recently reported the presence of a novel 32 kDa protein immunoreactive to a copper, zinc superoxide dismutase (SOD1) antibody within the spinal cord of patients with amyotrophic lateral sclerosis (ALS). This unique protein species was generated by biotinylation of spinal cord tissue extracts to detect conformational changes of SOD1 specific to ALS patients. To further characterize this protein, we enriched the protein by column chromatography and determined its protein identity by mass spectrometry. The protein that gave rise to the 32 kDa species upon biotinylation was identified as carbonic anhydrase I (CA I). Biotinylation of CA I from ALS spinal cord resulted in the generation of a novel epitope recognized by the SOD1 antibody. This epitope could also be generated by biotinylation of extracts from cultured cells expressing human CA I. Peptide competition assays identified the amino acid sequence in carbonic anhydrase I responsible for binding the SOD1 antibody. We conclude that chemical modifications used to identify pathogenic protein conformations can lead to the identification of unanticipated proteins that may participate in disease pathogenesis.

## 1. Introduction

Chemical modifications of proteins are useful in their applications to enhance and stabilize enzyme activities, cross-link different proteins, add tags for tracking and labeling proteins, and probe structural differences in protein conformations [1,2]. Protein modifications typically occur on amino acids side-chains that are accessible to the chemical reagents. A native protein acquires its conformation dependent upon its linear amino acid sequence and the local environment. Multiple conformations usually exist for a given protein as a regulatory mechanism for diverse physiological functions [3]. This variation in protein conformations also forms the basis for potential differences in the outcome of chemical modifications. For example, modifications of the sulfhydryl group of the cysteine residue often result in an increase in molecular mass of the protein that can be detected by immunoblot analysis [4]. It is also known that chemical modification can result in either loss or gain of immunoreactivity to specific antibodies [5,6].

Mutations in the human copper, zinc superoxide dismutase (SOD1) gene are responsible for approximately 2–5% of amyotrophic lateral sclerosis (ALS), an adult-onset neurological disease characterized by loss of motor neurons in the spinal cord as well as brainstem and motor cortex [7,8]. In an attempt to determine whether there are different SOD1 conformers associated with pathological state of ALS, we used biotinylation as a probe to detect potential conformational differences that can be observed with the SOD1 antibody by immunoblot and identified a novel 32 kDa immunoreactive species [9]. In this study, we identify carbonic anhydrase I (CA I) as the 32 kDa band detected by the anti-SOD1 antibody upon biotinylation of specific amino acids within CA I.

## 2. Materials and Methods

### 2.1. Human Samples

Human spinal cord autopsy samples were obtained from The Brain and Tissue Bank for Developmental Disorders of the National Institute of Child Health and Human Development (www.btbank.org, Baltimore, MA, US).

### 2.2. Protein Biotinylation

Cytosolic proteins from post-mortem tissue samples were prepared as described [9]. Protein concentrations were measured by the BCA method (Thermo Fisher Scientific, Pierce Protein Research Products, Rockford, IL). Biotinylation reaction was carried out as originally described [9]. Briefly, proteins were incubated with 10 mM Sulfo-NHS-LC-Biotin (Thermo Fisher Scientific) in PBS buffer, pH 7.4 for 25 min at 25 °C. The reaction was stopped by adding free lysine-HCl at a final concentration of 20 mM for 20 min at 25 °C. The control treatment was carried out in identical procedures except omitting Sulfo-NHS-LC-Biotin in the reaction.

### 2.3. Western Analysis

Proteins were separated on SDS-PAGE gels and transferred onto nitrocellulose membranes. Membranes were blocked in TBST, pH 7.4 containing 5% milk, before being incubated with a rabbit polyclonal anti-SOD1 antiserum [10] in the same buffer at 4 °C overnight. Membranes were washed with TBST and incubated with HRP-conjugated anti-rabbit IgG secondary antibody (Jackson ImmunoResearch Laboratories Inc., West Grove, PA) for 1 h. Subsequently, membranes were washed and visualized via ECL development (GE Healthcare Life Sciences, Piscataway, NJ, USA).

### 2.4. Ion Exchange Chromatography

A total of 200 μL of the cytosolic proteins was applied to a prepacked 5 mL anion exchange column (HiTrap Q HP, GE Healthcare Life Sciences) using the buffer of 20 mM Triethanolamine (TEA), pH 7.8. A total of 32 mL of the same buffer was used to wash the column after the sample loading. The flow-through materials were collected in 4 mL fractions (labeled as FT_1 through FT_8). The column was then eluted with 20 mM TEA containing 100 mM and 200 mM NaCl, respectively. A total of 12 mL elution buffer was used for each salt concentration and eluted proteins were collected in 4 mL fractions (labeled as 100_1, 2, 3 and 200_1, 2, 3, respectively). Each fraction was reduced to a final volume of 25 μL using 10 K NMWL centrifugal filter devices (Amicon Ultra-4, Millipore, Billerica, MA). An aliquot of each concentrated fraction was used for biotinylation and immunoblot analysis.

### 2.5. Purification of the 32 kDa Protein

The combined FT_2,3,4 fraction from a total of 800 μL of cytosolic proteins were separated on 10% SDS/PAGE and stained with SYPRO (Invitrogen, Carlsbad, CA). Protein bands of interests were excised and minced into small pieces. A total of 500 μL of the elution buffer (0.25 M Tris-HCl, pH 6.8/0.1% (w/v) SDS) was added and samples vortexed at 14,000 rpm for 30 min at room temperature. The procedure was repeated once to ensure the complete extraction of the protein. The supernatant were combined and concentrated via the MWCO-10K centrifugation unit (Amicon Ultra 4, Millipore). An aliquot of the eluted protein was biotinylated for the detection of the 32 kDa SOD1-IR.

### 2.6. Mass Spectrometry

The SYPRO-stained 32 kDa protein band was excised and processed for protein identification. Briefly, each gel slice was diced into small pieces (1 × 1 mm) and placed into an eppendorf tube. The gel was treated three times by adding 100 μL of 25 mM NH_4_HCO_3_/50% methanol, vortexing for 10 min, and removing the supernatant, followed by adding 50 μL of 10 mM DTT in 25 mM NH_4_HCO_3_ for incubation at 56 °C for 1 h and removing the supernatant. Subsequently, the gel was treated twice by adding 50 μL of 55 mM iodoacetamide for incubation in the dark for 45 min at room temperature, removing the supernatant and adding 100 μL of 25 mM NH_4_HCO_3_/50% methanol and vortexing for 10 min. Finally, the gel pieces were dehydrated by incubation with 50 μL of 50% acetonitrile (ACN) for 10 min and dried completely. Digestion of the protein was performed in 10 μL of 20 ng/μL trypsin solution and incubated overnight at 37 °C before transferring the digest solution into a clean 0.65 mL tube. 50 μL of 50% ACN/1% trifluoroacetic acid (TFA) was added to the gel pieces and vortexed for 30 min. The supernatant was transferred into the same tube. This step was repeated once before drying the gel completely. For MALDI-TOF, 3 μL of 50% ACN/1% TFA was added and vortexed for 1 min. 3 μL of saturated CHCA (a-cyano-4-hydroxycinammic acid) in 50% ACN/1% TFA was added and the suspension was spotted on MALDI target and let dry. Acquisition of peptide fingerprinting and MS/MS sequencing data were performed on Applied Biosystems ABI 4700 MALDI-TOF/TOF and Bruker Daltomics Ultraflex Ultimate Performance MALDI-TOF & TOF/TOF mass spectrometers.

Peaks were identified by peptide mass fingerprinting and sequenced by tandem MS/MS. Peptide and amino acid sequences were compared to Mascot mammalian protein database (www.matrixscience.com Version 2.2) to confirm identity using a maximum of 1 missed cleavage permission and 200 ppm measurement tolerance.

## 3. Results

### 3.1. Novel Immunoreactivity Recognized by Anti-SOD1 Antibody after Biotinylation

**Figure 1:**
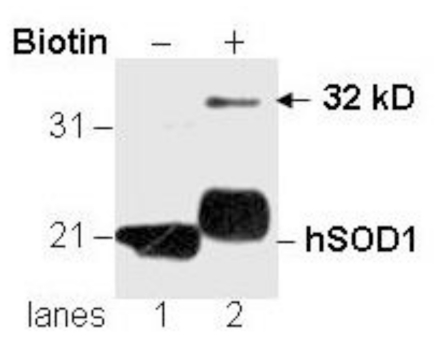
Novel immunoreactivity of the SOD1 antibody to a protein of 32 kDa after biotinylation. Spinal cord cytosolic proteins from an ALS subject in the absence (−) and presence (+) of biotin were analyzed by immunoblot analysis using the SOD1 antibody. Protein molecular weight markers are noted on the left. A protein band of 32 kDa became detectable only after biotinylation (indicated by the arrow).

Cytosolic proteins from spinal cords of ALS patients were treated with biotin followed by immunoblot analysis using the anti-SOD1 antibody (a rabbit polyclonal #354 antiserum against the peptide CYDDLGKGGNEESTK as previously described by Pardo *et al*. [10]). We generated the expected increased molecular mass to human SOD1 protein resulting from the addition of biotin moieties (Figure 1, lane 1 & lower band of lane 2). In addition, a SOD1-immunoreactive (SOD-IR) band of approximately 32 kDa was detected only after biotinylation (Figure 1, indicated by the arrow). Two potential explanations can account for this newly observed 32 kDa SOD1-IR species. One possibility is that biotinylated lysine residues within a different protein give rise to an epitope-like domain that is reactive with the anti-SOD1 antibody. Another possibility is that a covalent reaction between SOD1 and another protein occurred during the biotinylation and is responsible for the generation of the 32 kDa SOD1-IR species.

In addition, we tested multiple other anti-SOD1 antibodies including a different rabbit polyclonal antibody against the same peptide, polyclonal antibodies against different peptides in human SOD1 or against the entire protein, as well as monoclonal antibodies against human SOD1, none of which resulted in the same 32 kDa-IR band as seen with the #354 antiserum (data not shown). For the remainder of the experiments in this study, only #354 antiserum is used for the detection of the 32 kDa-IR band.

### 3.2. Purification and Identification of the Protein That Gives Rise to the 32 kDa-IR

**Figure 2:**
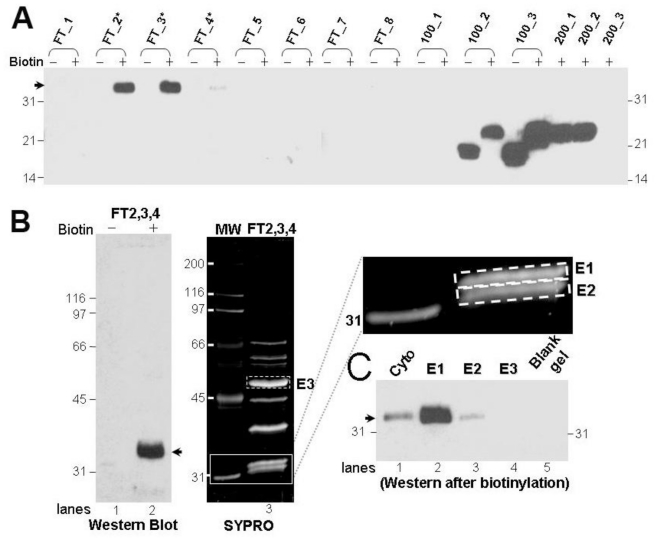
Purification of the protein species that gives rise to the 32 kDa-IR signal after biotinylation. All immunoblot analysis were performed with proteins either treated with (+) or without (−) biotin and detected by #354 antiserum. (**A**) The 32 kDa protein is partially purified in the flow-through (FT) fractions via anion exchange chromatography as described in Materials and Methods. A total of 200 μL of the cytosolic proteins from the SALS spinal cord were used. (**B**) Fractions from FT_2 to FT_4 were combined as FT2,3,4 and analyzed by both immunoblot analysis (left) and SYPRO staining (right) to visualize the proteins. Two protein bands were seen at the 32 kDa position by SYPRO (indicated by the solid rectangular box). The upper (E1) and lower (E2) band of the doublet, together with a strong protein band above the 45 kDa (E3), and a comparable size gel piece lacking any apparent protein bands by SYPRO, were excised, proteins eluted and treated by biotin, and analyzed via Western (**C**). The 32 kDa-IR signal is indicated by the arrow in (**C**). Lane 1 contains the biotinylated cytosolic protein from the SALS spinal cord as a positive control.

**Figure 3:**
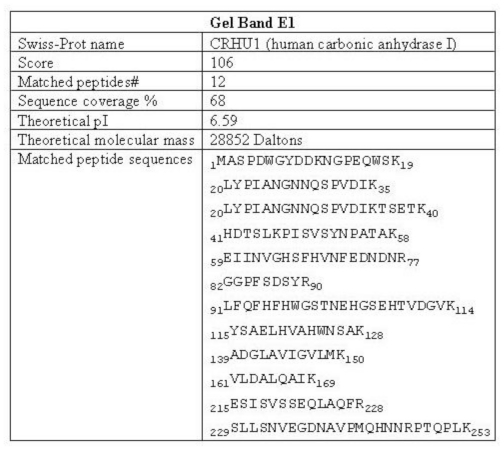
Identification of the purified protein as CA I. The E1 gel band from Figure 2 was digested with trypsin and subjected to amino acid sequencing by mass spectrometry. Details from the analyses used to identify band E1 as carbonic anhydrase I (CA I) are summarized. The protein score refers to a MASCOT search against the mammalian Swiss-Prot dataset with up to one missed tryptic cleavage site. A protein score greater than 68 is significant (p < 0.05). The matched peptide sequences, representing 68% of the CA I protein sequence, are shown.

In order to characterize the protein that generates the observed 32 kDa-IR, we purified the 32 kDa-IR signal by column chromatography. We found that anion exchange chromatography of the soluble tissue extract provided enrichment of a protein that produced the 32 kDa-IR upon biotinylation. The protein that generated the 32 kDa-IR was collected within column flow-through fractions FT_2 to FT_4 while all native molecular mass SOD1 was concentrated in the salt-eluted fractions (Figure 2A). This result indicates that the 32 kDa-IR signal was not generated from covalent linkage between an SOD1 molecule and another protein during the biotinylation reaction. The proteins in the combined fractions (FT_2 to FT_4, labeled as FT2,3,4) were separated and visualized by anti-SOD1 immunoblot as a single, broad 32 kDa band but via SYPRO staining were resolved as a protein doublet (Figure 2B, solid rectangular box). In order to determine which band in the doublet gave rise to the 32 kDa-IR signal, both bands were excised individually from the gel, eluted, biotinylated, and immunolabeled with #354 antiserum. We determined that the upper gel (labeled E1) is the protein that upon biotinylation gave rise to the immunoreactivity to #354 antiserum (Figure 2C). This same band was further processed for peptide fingerprinting and amino acid sequencing via MALDI-TOF/TOF mass spectrometry as described in Section 2. Amino acid sequence information from 12 peptides generated by tryptic digestion demonstrated that carbonic anhydrase I (CA I) is the protein in gel band E1, with 68% sequence coverage (Figure 3).

### 3.3. Human CA I Is Necessary and Sufficient to Give Rise to the 32 kDa-IR Species upon Biotinyation

**Figure 4:**
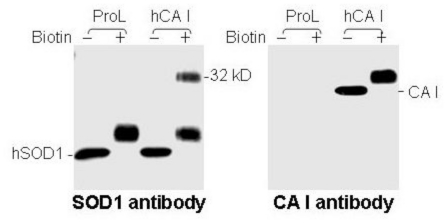
Human CA I is necessary and sufficient for 32 kDa-IR species to #354 antiserum upon biotinylation. Immunoblot analysis of proteins from HEK cells transiently transfected with human CA I (hCA I) or prolactin (ProL) treated without (−) or with (+) biotin. Identical samples were used for two blots which were probed with #354 antiserum (left panel) and anti-CA I (right panel) antibodies, respectively.

To further determine whether human CA I is responsible for generating the 32 kDa-IR species upon biotinylation, we tested the ability of human CA I to generate the 32 kDa-IR in cultured cells. HEK cells do not express detectable levels of endogenous CA I, but after transfection with a plasmid containing human CA I we detected a 30 kDa immunoreactive band that also increased in molecular mass to 32 kDa after biotinylation by an anti-CA I antibody (Figure 4, right panel). In contrast, transfection with a control plasmid containing prolactin (ProL) failed to generate any anti-CA I immunoreactive bands (Figure 4, right panel). Furthermore, only HEK cells expressing human CA I, but not ProL, exhibited the 32 kDa-IR band upon biotinylation, in addition to endogenous native 20 kDa hSOD1 when probed by the #354 antiserum (Figure 4, left panel)

We also investigated whether endogenous mouse CA I was capable of generating the same 32 kDa-IR species upon biotinylation, as there is an overall 78% homology between human and mouse CA I proteins. Mouse spinal cord tissues from non-transgenic as well as multiple SOD1-transgenic mice were examined and the 32 kDa-IR signal was not detected by the #354 antiserum (Supplementary data).

The above results demonstrate that only human CA I is both necessary and sufficient to generate the 32 kDa-IR species upon biotinylation.

### 3.4. Biotinylation of an Epitope-Like Sequence in Human CA I Resulted in the Novel Immunoreactivity

**Figure 5:**
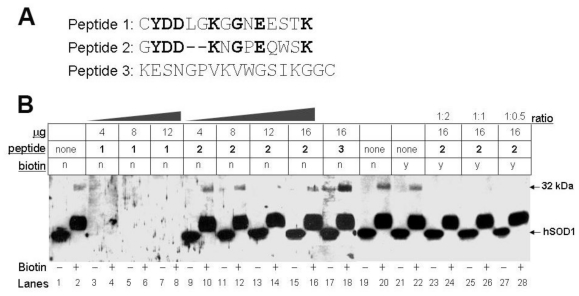
A biotinylated epitope-like sequence in human CA I is responsible for the novel immunoreactivity to #354 antiserum. (**A**) Sequences and alignment of the three peptides used in the peptide competition experiments. Identical amino acids were indicated in bold. (**B**) Peptide competition experiments were carried out by immunoblot analysis of cytosolic proteins from an ALS spinal cord in the absence (−) or presence (+) of biotin using #354 antiserum. Multiple blots with identical sample preparations were used for incubations with the antibody: (i) alone; (ii) pre-absorbed with different amounts of peptides; or iii) pre-absorbed with biotinylated peptides. The labels for the table above the blot: “μg”: the amount of the peptide used; “peptide”: either no peptide (as “none”) or which peptide (as “1”, “2”, or “3”) was used in pre-absorption with the antibody; “biotin”: whether the peptide was biotinylated or not (as “y” or “n“, respectively); “ratio”: molar ratio of the peptide to biotin.

To further understand the mechanism by which CA I obtained immunoreactivity to #354 antiserum upon biotinylation, we performed peptide competition experiments using three different peptides (Figure 5A). Peptide 1 is a portion of the sequence used to generate the anti-SOD1 antibody. Peptide 2 is a sequence identified within CA I that exhibits 50% sequence identity to peptide 1 as determined by SIM alignment tool [11], and peptide 3 is an unrelated sequence used as a negative control. The identical amino acids between peptide 1 & 2 are indicated by bold letters, including two lysine (K) residues that can be biotinylated. As expected, peptide 1 competed and eliminated the immunoreactivity for both the native 20 kDa hSOD1 and the 32 kDa-IR species (Figure 5B, lanes 3–8), as it occupied the epitope-recognition site in the antibody. In contrast, peptide 2 did not compete either immunoreactive species, while biotinylated peptide 2 competed only the 32 kDa-IR species (Figure 5B, lanes 9–16, 23–28). This data demonstrated that peptide 2 does not interact with the epitope-recognition site in the antibody, but upon biotinylation it could occupy the site. Peptide 3 that lacks homology to either peptide 1 or 2 failed to compete #354 antiserum recognition on either the two immunoreactive signals (Figure 5B, lanes 17–18). Together, these data further indicate that biotinylated amino acids within peptide 2 of CA I give rise to the 32 kDa-IR.

## 4. Conclusions

Although it is known that chemical modifications of amino acid side-chains in the protein can alter immunoreactivity to antibodies, most involve either an increase or loss of affinity to the antibodies against that particular protein [5] or a closely related protein [6]. Protein biotinylation generating novel immunoreactivity to an antibody to an unrelated protein is uncommon. In this report, we elucidated the mechanism by which such phenomenon can occur by detecting biotinylated proteins using immunoblot analysis with the SOD1 antibody #354.

The results of this study demonstrate that protein modifications can potentially generate novel conformational features that may be detected by antibodies to disparate proteins. Our specific example identifies a peptide sequence in human CA I that upon biotinylation, is sufficient and necessary to be recognized by a polyclonal anti-SOD1 antibody generated against a SOD1-specific peptide sequence (#354 polyclonal antiserum). This certainly is unexpected and rather an uncommon event. In fact, the 32 kDa species was suggested to be an SOD1-dimer which contained SOD1 protein sequences when analyzed in a non-purified form [9]. By further purification of the protein, we demonstrate here that human CA I, a protein unrelated to SOD1, is responsible for generating a biotinylated species that is recognized by the SOD1 antibody.

This study is important in the following two aspects. First, we have identified a molecular mechanism underlying newly gained epitope recognition to an antibody of an unrelated protein due to chemical modifications of amino acid side-chains. The molecular basis is the peptide sequence in human CA I which shares 50% homology to the epitope sequence in human SOD1 that was used to generate the #354 polyclonal antiserum. This peptide sequence homology is not sufficient to result in a cross-reaction of the CA I protein with the anti-SOD1 antibody by traditional immunoblot analysis. In addition to the amino acid sequence homology, the potential electrical charges that are imparted by lysine and glutamic acid residues can serve as a barrier to the antibody recognition if a hydrophobic interaction is involved. However, biotinylation of the CA I protein within this peptide sequence generates an epitope-like structure within CA I that becomes immunoreactive to #354 antiserum. Therefore, features other than high homology within the amino acid sequence must be considered when evaluating the basis of peptide antibody recognition, especially if chemical modifications are performed to the protein. What are the potential explanations for #354 polyclonal antiserum to react to both biotinylated CA I as well as the SOD1 epitope? The answer is either the two epitope recognition sites are in the same linear sequence of amino acids or that biotinylation of CA I generates a conformational epitope that is now recognized by the anti-SOD1 antibody. We know that biotinylated CA I peptide cannot block the SOD1-recognition site while the SOD1 peptide can block both recognition sites. Further studies are required to address this issue. However our results do suggest that careful characterizations of the nature of antibody and protein recognition is warranted in experimental investigations, especially those involving alterations of protein side-chain structures such as the biotinylation approach used in our studies [9].

Protein identification via mass spectrometry determined that CA I is the molecule that acquires immunoreactivity to anti-SOD1 antibody upon protein biotinylation. Recent studies have demonstrated that antibodies against the P2X4 subunit of the ATP receptor recognize misfolded mutant G93A SOD1 in a transgenic mouse model of ALS [12], indicating that protein misfolding may also induce antibody cross reactivity.

Our study has identified a protein, not previously implicated in ALS, in an unbiased albeit limited way by use of a specific SOD1 antibody. CA I is a cytosolic protein known to function in carbon dioxide transport within cells and at the level of mitochondria [13], and its carbonic anhydrase activity contributes to maintaining cellular pH homeostasis [14]. Inhibition of CA I can reduce muscle contraction and calcium storage capacity [15], and increased plasma levels of carbonic anhydrase III has been detected in patients with neuromuscular disorders [16]. CA I is an abundant protein in blood and can also be detected in cerebrospinal fluid (CSF) (Liu *et al*., unpublished result). Initial screenings of CA I from either blood or CSF samples from normal and ALS patients did not reveal observable differences between the two populations (Liu *et al*. unpublished results). However, characterization of neuronal CA I has not been reported and awaits future studies. While the contribution of CA I to ALS pathology and disease pathogenesis remains unclear, further exploration of CA I in motor neuron disease is warranted and may lead to new therapeutic opportunities for ALS.

## Supplementary data

**Figure S1:**
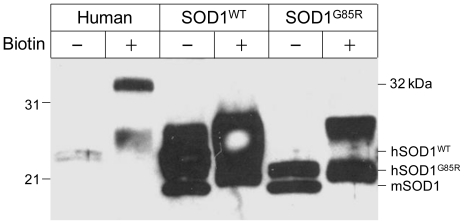
The 32 kDa-IR species is not detected in the spinal cord of SOD1-linked transgenic mice. Cytosolic proteins from the spinal cords of transgenic mice (SOD1^WT^ and SOD1^G85R^, containing the human wild-type and G85R mutant SOD1s, respectively) were treated with (+) or without (−) biotin and probed with the #354 anti-SOD1 antiserum via immunoanalysis as described in Materials & Methods. A positive control from a human sample for the 32 kDa-IR species was included (Human). mSOD1: endogenous mouse SOD1. Molecular weight markers are indicated on the left. Biotinylated mSOD1 and hSOD1 are both up-shifted in the presence of biotin.
